# Improving chemical entity recognition through h-index based semantic similarity

**DOI:** 10.1186/1758-2946-7-S1-S13

**Published:** 2015-01-19

**Authors:** Andre Lamurias, João D Ferreira, Francisco M Couto

**Affiliations:** 1 LaSIGE, Departamento de Informática, Faculdade de Ciências, Universidade de Lisboa, 1749-016 Lisboa, Portugal

**Keywords:** Ontologies, Semantic Similarity, Named Entity Recognition, ChEBI

## Abstract

**Background:**

Our approach to the BioCreative IV challenge of recognition and classification of drug names (CHEMDNER task) aimed at achieving high levels of precision by applying semantic similarity validation techniques to Chemical Entities of Biological Interest (ChEBI) mappings. Our assumption is that the chemical entities mentioned in the same fragment of text should share some semantic relation. This validation method was further improved by adapting the semantic similarity measure to take into account the h-index of each ancestor. We applied this method in two measures, simUI and simGIC, and validated the results obtained for the competition, comparing each adapted measure to its original version.

**Results:**

For the competition, we trained a Random Forest classifier that uses various scores provided by our system, including semantic similarity, which improved the F-measure obtained with the Conditional Random Fields classifiers by 4.6%. Using a notion of concept relevance based on the h-index measure, we were able to enhance our validation process so that for a fixed recall, we increased precision by excluding from the results a higher amount of false positives. We plotted precision and recall values for a range of validation thresholds using different similarity measures, obtaining higher precision values for the same recall with the measures based on the h-index.

**Conclusions:**

The semantic similarity measure we introduced was more efficient at validating text mining results from machine learning classifiers than other measures. We improved the results we obtained for the CHEMDNER task by maintaining high precision values while improving the recall and F-measure.

## Background

Named entity recognition (NER) is the text mining task of automatically identifying the entities mentioned in scientific articles, patents, and other text documents. The BioCreative challenge is a community effort to evaluate text mining and information extraction systems applied to the biological domain. One of the tasks proposed for the fourth edition of this competition was the chemical compound and drug named entity recognition (CHEMDNER) task. It was essentially a NER task for detecting chemical compounds and drugs in MEDLINE documents, in particular those that can be linked to a chemical structure [1]. The task organizers provided a training corpus composed of 10,000 MEDLINE titles and abstracts that were manually annotated by domain experts.

Measuring the semantic similarity between the chemical entities mentioned in a given fragment of text has been shown to provide an effective validation method to achieve high precision values [2]. Our assumption is that entities mentioned in the same fragment of text share some semantics between them, thus entities with low semantic similarity are considered to be errors. To each recognized term, we associate a chemical entity in Chemical Entities of Biological Interest (ChEBI) ontology [3], and calculate a validation score based on its similarity to other terms. The purpose of this method is to filter out false positives incorrectly classified as relevant chemical entities by the machine learning classifiers.

Many Semantic Similarity Measures (SSM) rely on the notion of Information Content (IC) of a concept to account for its specificity [4,5]. IC measures can be extrinsic (relying on an external corpus to quantify the specificity of a concept) as in [4] but the need for the external corpus has been shown to be a disadvantage. In fact, recent studies have shown that intrinsic IC measures, which depend only on the structure of the ontology, are comparable to extrinsic measures [6].

In this manuscript we present the approach we used for the CHEMDNER task and its recent improvements. Our system was built on the ICE framework [7] and we applied semantic similarity for validating the results and therefore achieve high levels of precision. This validation process was improved by using a SSM that takes into account only the most relevant ancestors of a concept. We used the h-index to measure its concept relevance in the ontology, adapting from the definition proposed by Hirsch [8] to measure the impact of the research work of a scientist.

The rest of this paper is organized as follows: Results and discussion presents the results we obtained with our original system, and with the improvements we implemented post-challenge, using cross-validation; Conclusions summarizes the main conclusions from this work; Methods describes the approach we used for the competition and how we improved it since then using an h-index based semantic similarity measure.

## Results and discussion

### Task description

#### CHEMDNER corpus

The CHEMDNER corpus consists of 10,000 MEDLINE titles and abstracts and was originally partitioned randomly in three sets: training, development and test [9]. The chosen articles were sampled from a list of articles published in 2013 by the top 100 journals of a list of categories related to the chemistry field. These articles were manually annotated by a team of curators with background in chemistry. Each annotation consisted of the article identifier, type of text (title or abstract), start and end indices, the text string and the type of chemical entity, which could be one of the following: trivial, formula, systematic, abbreviation, family and multiple. There was no limit for the number of words that could refer to a CEM but due to the annotation format, the sequence of words had to be continuous. There were a total of 59,004 annotations on the training and development sets, which consisted of 7,000 documents.

#### CEM and CDI subtasks

There were two types of predictions the participants could submit for the CHEMDNER task: a ranked list of unique chemical entities described on each document, for the Chemical Document Indexing (CDI) subtask, and the start and end indices of each chemical entity mentioned on each document for the Chemical Entity Mention (CEM) subtask. Using the CEM predictions, it was possible to generate results for the CDI subtask, by excluding multiple mentions of the same entity in each document. A gold standard for both subtasks was included with the corpus, which could be used to calculate precision and recall of the results, with the evaluation script released by the organization. Each team was allowed to submit up to five different runs for each subtask.

### Submission for the CHEMDNER task

Using different combinations of the developed methods, five runs were submitted for each subtask by our team. Each run used different corpora and different validation methods. We used the CHEMDNER corpus and two external corpora for run 3, while only the CHEMDNER corpus was used for run 3*. These two runs provide the maximum recall we can achieve, since no validation process was employed. Run 3* was not submitted to the competition since the recall obtained with run 3 was higher. Run 2 uses only the CHEMDNER corpus and a high validation threshold based on the CRF confidence, ChEBI mapping score and semantic similarity to other entities in the same document. These three values were also used to train a Random Forest classifier to validate the CRF results, which corresponds to run 1. Run 4 uses only the CHEMDNER corpus, like run 3*, but each result is validated with semantic similarity, while run 5 uses the same training corpora as run 3, but also with the semantic similarity validation. Each run is described with more detail in the Methods section.

With the results from each run, we were able to generate predictions for the CEM subtask, using every entity recognized, and for the CDI subtask, considering only unique entities for each document. The metrics for each set of predictions were calculated using the official evaluation script on the results of 3-fold cross-validation for the CHEMDNER training and development dataset (Table 1). The official evaluation results are presented in Table 2. We can observe that generally, the results for the test set are better than using cross-validation.

**Table 1:** Precision (P), Recall (R) and F-measure (F) estimates for each method used, using cross-validation, for the Chemical Documents Indexing task (CDI) and Chemical Entity Mention task (CEM).

Run	CDI			CEM		
	P	R	F	P	R	F
1	84.1%	72.6%	77.9%	87.3%	70.2%	77.8%
2	95.0%	6.5%	12.2%	95.0%	5.9%	11.1%
3	52.1%	80.4%	63.3%	57.1%	76.6 %	65.4%
3*	76.7%	75.7%	76.2%	80.2%	72.8 %	76.3%
4	87.9%	22.7%	36.1%	89.7%	21.2%	34.3%
5	87.8%	22.7%	36.1%	79.9%	22.6%	35.3%

**Table 2:** Precision (P), Recall (R) and F-measure (F) obtained with the test set, for the Chemical Documents Indexing task (CDI) and Chemical Entity Mention task (CEM).

Run	CDI			CEM		
	P	R	F	P	R	F
1	85.3%	68.9%	76.2%	87.8%	65.2%	74.8%
2	96.8%	8.06%	14.9%	96.7%	7.11%	13.3%
3	57.7%	81.5%	67.5%	63.9%	77.9 %	70.2%
4	91.9%	24.4%	38.6%	92.9%	22.7%	36.4%
5	77.1%	27.3%	40.3%	79.7%	25.0%	38.1%

The results of runs 3 and 3* show the performance of our system using no validation process. The values obtained are comparable with other applications of Mallet to this same task, for example, [10]. Since run 3 uses external corpora, the precision is much lower than run 3*, which uses only the CHEMDNER corpus. With each validation process, corresponding to the other four runs, we were able to improve precision, while run 1 also improved the F-measure of the CEM task by 4.6% on the test set. Every validation process also lowered significantly our recall, between 12%-60%. For this reason, we focused our work on improving the validation process so that the effect on recall is reduced.

Comparing with the results from other teams, we achieved high precision values, especially on run 2 (96.8% for the CDI task), which was the second highest of all teams. However, the recall obtained with that run was also one of the lowest of the competition. These results should be viewed as an extreme case for our validation process, since too many true positives were wrongly filtered out from the final result. Using semantic similarity (run 4), we also achieved high precision, without lowering the recall as much as run 2. The validation methods used should be improved so that we can still obtain high precision values with minimal effect on the recall.

### Results using h-index

**Figure 1:**
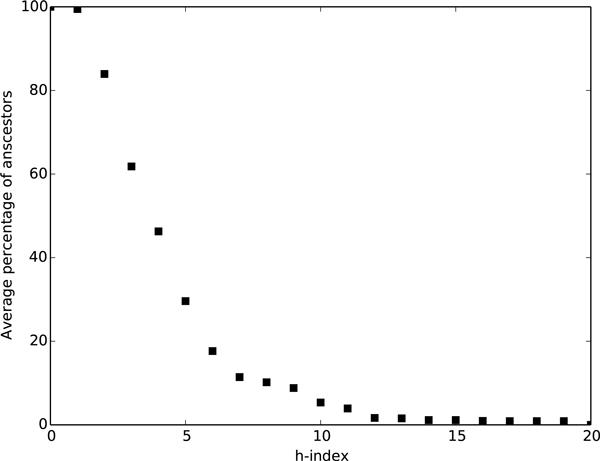
**Average percentage of ancestors discarded using each h-index value**.

We computed the h-index of each class in the ChEBI ontology. Figure 1 shows the average percentage of ancestors with an h-index above each threshold. We can see that about 10% of ancestors have an h-index higher than 7; based on this results, we decided to use our proposed measure with h-index of 2, 3, 4, 5 and 6. This decision was further validated when the results in Table 3 were obtained. In fact, once we use an h-index threshold of 6, precision values start to decrease, suggesting that the SSM scores start to degrade because of the high amount of concepts removed from the ancestry.

**Table 3:** Precision values obtained with each SSM for a fixed recall.

	P	R
simUI	92.97%	20.31%
simUI_2_	93.14%	20.23%
simUI_3_	93.01%	19.73%
simUI_4_	93.10%	19.77%
simUI_5_	93.35%	19.81%
simUI_6_	93.00%	20.16%

simGIC	92.95%	20.23%
simGIC_2_	93.14%	20.23%
simGIC_3_	93.23%	19.85%
simGIC_4_	93.24%	20.09%
simGIC_5_	93.19%	20.10%
simGIC_6_	93.10%	19.79%

We tested each measure for different validation thresholds, obtaining different precision and recall values for each threshold and each SSM. As we increase the validation threshold, ideally the precision should increase without affecting the recall. Eventually, true positives are also eliminated by this process, lowering the recall as the validation threshold increases.

**Figure 2:**
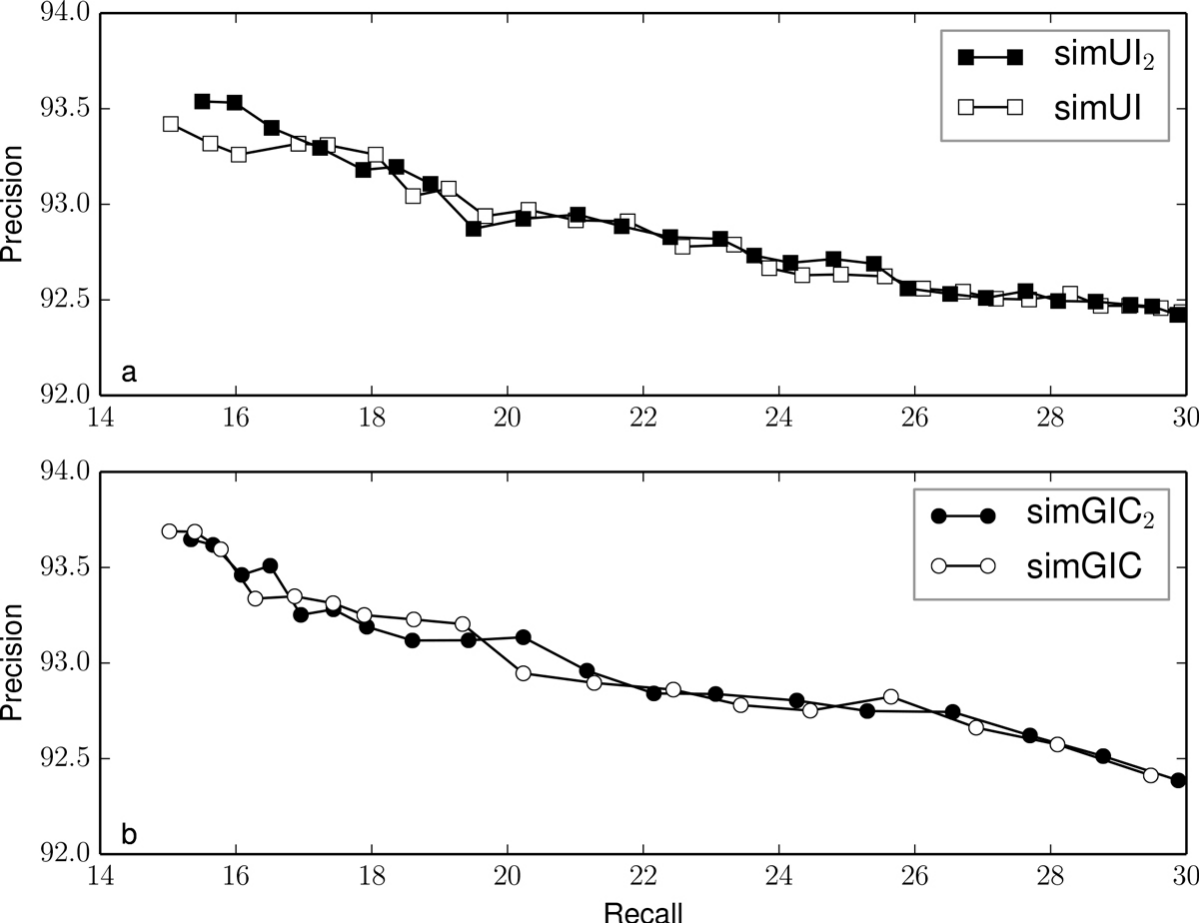
**Comparison of precision and recall values for different thresholds between simUI and simGIC and variants with h-index ≥ 2**.

**Figure 3:**
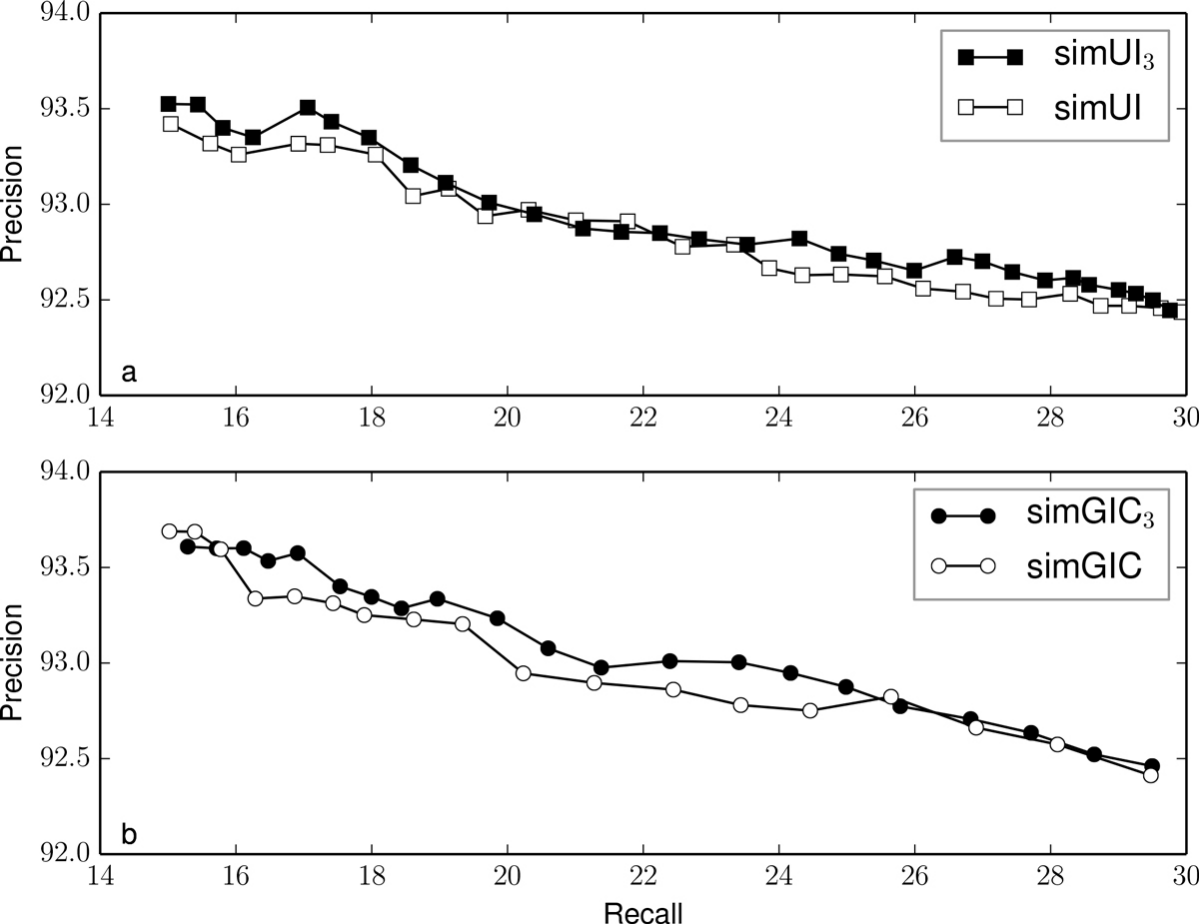
**Comparison of precision and recall values for different thresholds between simUI and simGIC and variants with h-index ≥ 3**.

**Figure 4:**
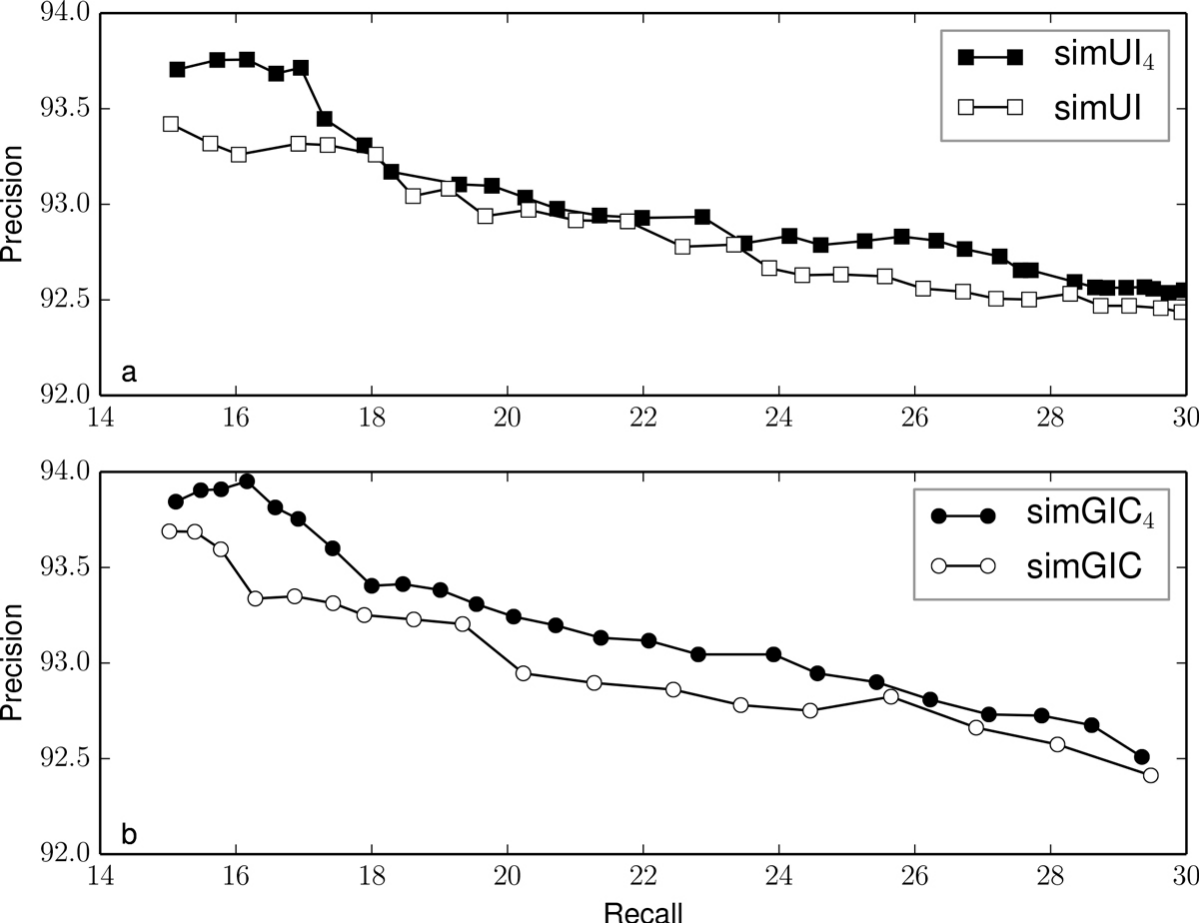
**Comparison of precision and recall values for different thresholds between simUI and simGIC and variants with h-index ≥ 4**.

**Figure 5:**
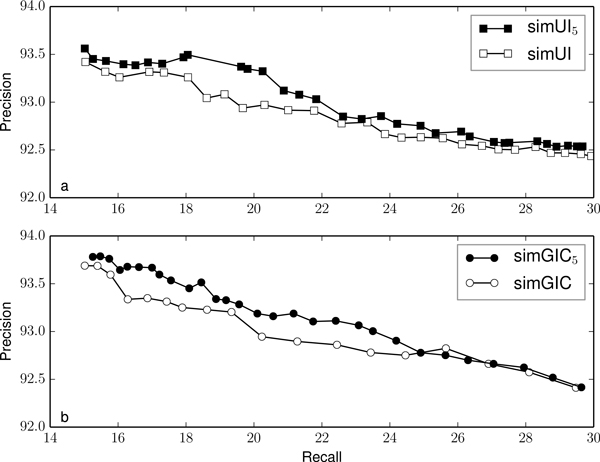
**Comparison of precision and recall values for different thresholds between simUI and simGIC and variants with h-index ≥ 5**.

**Figure 6:**
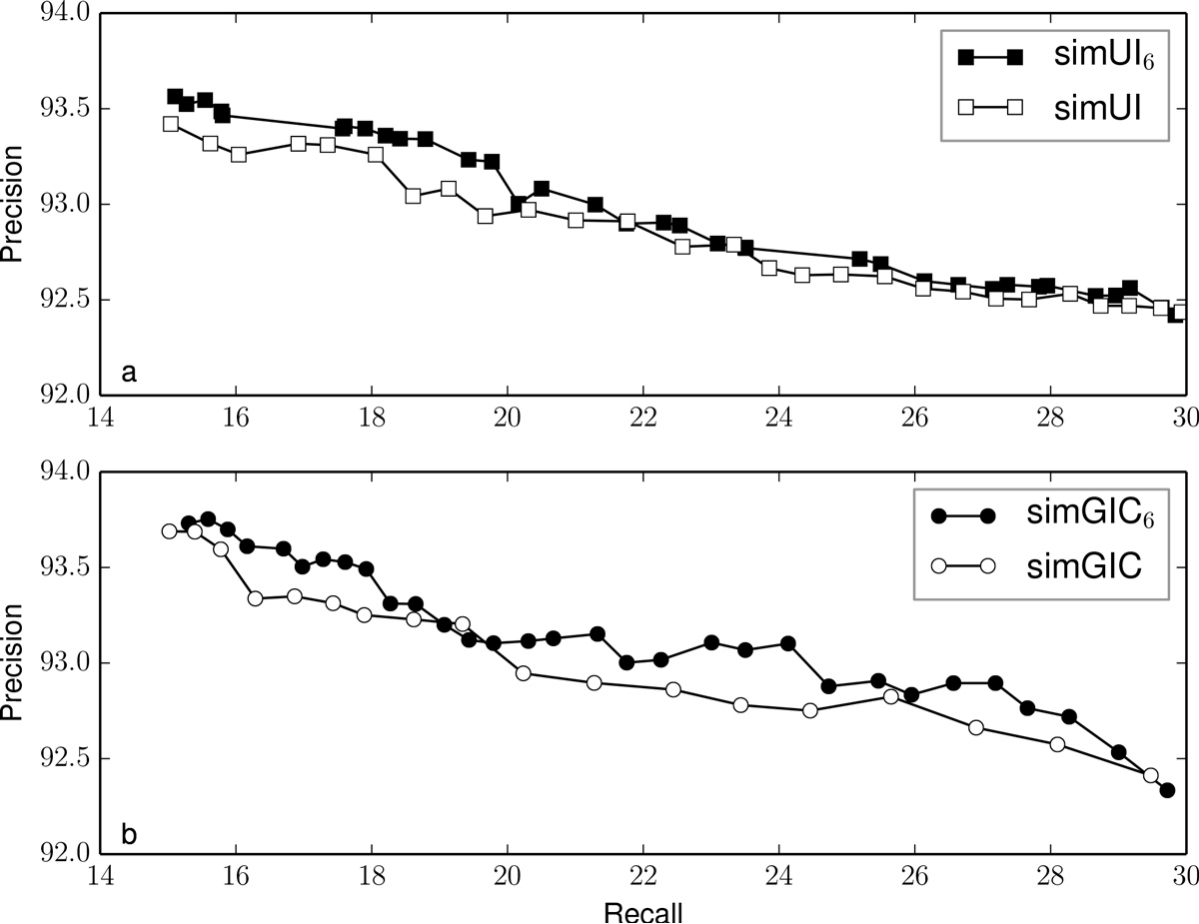
**Comparison of precision and recall values for different thresholds between simUI and simGIC and variants with h-index ≥ 6**.

Figures 2, 3, 4, 5 and 6 compare the precision and recall values obtained for different validation thresholds between simUI and simGIC and our proposed measure with five different h-index values. We restricted the recall values between 15% and 30%, since this is where the most of the points lie. Using our proposed measures, we obtained generally higher precision values for the same recall. This indicates that using the h-index information to measure semantic similarity results in a better performance at filtering out false positives from machine learning results. Furthermore, as the h-index increases, the difference between the original and our proposed measure increases. While on Figure 2, points are mostly overlapping, this is less frequent on Figure 3, as the h-index measure achieves higher precision values. Between Figures 5 and 6, this difference is less noticeable, which indicates that for higher h-index values, the filter becomes less efficient.

To confirm that the new adapted measures performed better at excluding fewer true positives, we compared the precision value obtained for each measure, with a fixed recall of 20%, on table 3. We selected the points from Figures 2, 3, 4, 5 and 5 that were closest to a recall of 20%. Between each measure, the precision correspondent to similar recall values improves with the h-index used for the measure.

## Conclusions

In this paper we proposed a novel method to compute the semantic similarity between chemical entities, to improve the chemical entity identification in texts. This method is based on the h-index, which we computed in the ChEBI ontology. By using the h-index to improve the simUI and simGIC measures, we were able to filter out fewer true positives with our validation process, and achieve higher precision values for the same recall. Comparing the simGIC with the simUI measure, which does not take into account the information content, the former measure achieved better results. The improvement is relatively small, but this may be because the NER applied was already well tuned for precision. This is an indication that the h-index provides a good estimate for the relevance of a class for the computation of the semantic similarity between two classes.

In the future we intend to study the effect of other parameters in our system to achieve a better balance between recall and precision, using the results obtained with the testing runs as a starting point. The semantic similarity measure used for filtering can be further improved by incorporating the disjointness information which has been shown to improve the accuracy of semantic similarity values [11]. Furthermore, these values can also be improved by matching the ChEBI entities with other ontologies and incorporating the semantic similarity values from those ontologies [12]. While the basic framework of our system is currently available online http://www.lasige.di.fc.ul.pt/webtools/ice/, we will update this web tool with the work described in this paper, as well as with a module capable of extracting interactions described between chemical entities.

## Methods

### Systems description for the CHEMDNER task

#### Datasets used

In addition to the provided CHEMDNER dataset, for training our classifiers, we used the DDI corpus dataset provided for the SemEval 2013 challenge [13], and a patent document corpus released publically by the ChEBI team [14]. The DDI dataset contains two sub-datasets, one that consists of MEDLINE abstracts, and the other of DrugBank descriptions. All named chemical entities were labeled with their type which could be one of the following: Drug (generic drug names), Brand (brand names), Group (mention of a group of drugs with a common property) and Drug_n (substances not approved for human use). Based on this label, we created four datasets from the DDI corpus dataset, each containing only one specific type of annotated entities. Considering the seven types of chemical entities of the CHEMDNER corpus, we also created seven datasets from this corpus.

#### CRF entity recognition

For this competition, we used the implementation of Conditional Random Fields (CRFs) on Mallet [15], with the default values. In particular, we used only an order of 1 in the CRF algorithm. The following features were extracted from the training data to train the classifiers:

**Stem: **Stem of the word token with the Porter stemming algorithm

**Prefix and Suffix size 3: **The first and last three characters of a word token.

**Number: **Boolean that indicates if the token contains digits.

Furthermore, each token was given different label depending on whether it was not a chemical entity, a single word chemical entity, or the start, middle or end of a chemical entity.

Since Mallet does not provide a confidence score for each label, we had to adapt the source code based on suggestions provided by the developers, so that for each label, a probability value is also returned, according to the features of that token. This information was useful to adjust the precision of our predictions, and to rank them according to how confident the system is about the extracted mention being correct.

We used the provided CHEMDNER corpus, the DDI corpus and the patents corpus for training multiple CRF classifiers, based on the different types of entities considered on each dataset. Each title and abstract from the test set was classified with each one of these classifiers. In total, our system combined the results from fourteen classifiers: eight trained with the CHEMDNER corpus (7 types + 1 with every type), five trained with the DDI corpus (4 types + 1 with every type) and one trained with the patents corpus.

After participating in the BioCreative IV challenge, we implemented a more comprehensive feature set with the purpose of detecting more chemical entities that would be missed by a smaller feature set. These new features are based on orthographic and morphological properties of the words used to represent the entity, inspired by other CRF-based chemical named entity recognition systems that had also participated in the challenge [16-19,10]. We integrated the following features:

**Prefix and Suffix sizes 1, 2 and 4: **The first and last n characters of a word token.

**Greek symbol: **Boolean that indicates if the token contains Greek symbols. Case pattern: "Lower" if all characters are lower case, "Upper" if all characters are upper case, "Title" if only the first character is upper case and "Mixed" if none of the others apply.

**Word shape: **Normalized form of the token by replacing every number with '0', every letter with 'A' or 'a' and every other character with 'x'.

**Simple word shape: **Simplified version of the word shape feature where consecutive symbols of the same kind are merged.

**Periodic Table element: **Boolean that indicates if the token matches a periodic table symbols or name.

**Amino acid: **Boolean that indicates if the token matches a 3 letter code amino acids.

With these new features, we were able to achieve better recall values while maintaining high precision. However, only the original list of features was used for the BioCreative IV challenge.

#### ChEBI resolution

After having recognized the named chemical entities, our method performs their resolution to the ChEBI ontology. The resolution method takes as input the string identified as being a chemical compound name and returns the most relevant ChEBI identifier along with a mapping score.

To perform the search for the most likely ChEBI entity for a given entity we employed an adaptation of FiGO, a lexical similarity method [20]. Our adaptation compares the constituent words in the input string with the constituent words of each ChEBI entity, to which different weights have been assigned according to its frequency in the ontology vocabulary. A mapping score between 0 and 1 is provided with the mapping, which corresponds to a maximum value in the case of a ChEBI entity that has the exact name as the input string.

Our resolution method was applied to the named chemical entities on the CHEMDNER training and development sets. We were able to find a ChEBI identifier for 69.2 % of these entities. The fraction of entities our method was unable to resolve for each type is shown in Table 4.

**Table 4:** Number of chemical entities from the CHEMDNER corpus not mapped to ChEBI.

Type	Systematic	Identifier	Formula	Trivial	Abbreviation	Family	Multiple
Unmapped	3382	1156	3972	3622	4181	1690	91
	(25.1%)	(88.2%)	(46.3%)	(20.3%)	(46.2%)	(20.3%)	(23.3%)
Total	13472	1311	8585	17802	9059	8313	390

#### Filtering false positives with a Random Forest model

With the named chemical entities successfully mapped to a ChEBI identifier, we were able to calculate Gentleman's simUI [21] for each pair of entities on a fragment of text. This measure is a structural approach, which explores the directed acyclic graph (DAG) organization of ChEBI [22]. We then used the maximum semantic similarity value for each entity as a feature for filtering and ranking.

The output provided for each putative chemical named entity found is the classifier's confidence score, and the most similar putative chemical named entity mentioned on the same document through the maximum semantic similarity score. Using this information, along with the ChEBI mapping score, we were able to gather 29 features for each prediction. When a chemical entity mention is detected by at least one classifier, but not all, the confidence score for the classifiers that did not detect this mention was considered to be 0. These features were used to train a classifier able to filter false positives from our results, with minimal effect on the recall value. We used our predictions obtained by cross-validation on the training and development set to train different Weka [23] classifiers, using the different methods implemented by Weka. The method that returned better results was Random Forest, and so we used that classifier on our test set predictions.

#### Post-processing

Some simple rules were also implemented in an effort to improve the quality of the annotations:

1 Exclude if one of the words is in a stop words list

2 Exclude text with no alphanumeric characters

3 Delete the last character if it is a dash ("-")

A list of common English words was used as stop words in post-processing. If a recognized chemical entity was part of this list or one of the words on the list was part of the chemical entity, then we assumed that it was a recognition error and should be filtered out and not be considered a chemical entity. This list was tuned with the rules used on the annotations of the gold standard. The other rules were implemented after analyzing common errors made by the CRF classifiers.

#### Testing Runs

We used different combinations of training corpora and validation processes for each run (see Table 5), but the basic pipeline was constant: (i) recognition of the chemical entities with CRF, and (ii) validation of each entity by mapping to ChEBI and computing semantic similarity.

**Table 5:** Corpora and validation methods used for each run.

	Corpora		Validation		
Run	CHEMDNER	DDI/PAT	SSM	COMBINED	RF
1	X	X			X
2	X			X	
3	X	X			
3*	X				
4	X		X		
5	X	X	X		

Different runs use different corpora for the CRF step: each uses (1) either the CHEMDNER corpus by itself or (2) the CHEMDNER corpus along with the DDI and patents (PAT) corpora. DDI and PAT were not annotated with the same criteria used for the CHEMDNER corpus, and do not contain the same type of texts. The DDI corpus is focused on drug names and contains drug interaction descriptions and PubMed abstracts, while PAT contains only patents annotated with chemical named entities.

For the validation process, we used three different methodologies: (1) The first methodology was to map the recognized entities to ChEBI and then apply the semantic similarity measure described previously to filter the entities based on a fixed threshold (SSM). (2) The second approach was to combine the confidence scores obtained with Mallet and ChEBI mapping score with the SSM values for each entity, computing a new score which was also used to filter the CRF results based on a threshold (COMBINED). (3) Finally, we used the three scores independently to produce a Random Forest to classify each entity as a true positive or a false positive (RF).

Experimenting with cross-validation on the training and development sets, we assembled different combinations of these methods (see Table 5).

On run 1, we use the full set of corpora alongside a RT validation. This was done after noticing that the Random Forest classifiers provides a better balance between precision and recall than a simple approach based on a score and threshold (approaches SSM and COMBINED). Furthermore, using every corpus provided more features, which is generally beneficial in CRF (Run 1).

For run 2, we used only the CHEMDNER corpus and the COMBINED validation process, since the combined score of each entity is more detailed than just one of the values. We determined empirically the threshold of 0.8 for this run, which gave us our maximum precision value.

Run 3 is equivalent to a baseline. In fact, this run uses only the results obtained with a CRF classifier trained with the full set of corpora, without a validation step. To better understand the effect of the training corpus, we also created a run 3*, where the CRF was trained with the CHEMDNER corpus only. The results of these two runs (3 and 3*) establish in fact the maximum recall value that can be expected with our approach, as they result in a non-filtered list which the validation step trims down. In fact, the perfect validation step should be able to remove from the CRF results all the false positive recognitions, but can never increase the number of correctly recognized entities. Notice that run 3* was not submitted for evaluation at the BioCreative contest, as only 5 runs were allowed.

Runs 4 and 5 use the SSM validation step, along with either the CHEMDNER corpus alone (run 4) or the full set of corpora (run 5) This selection was done in order to evaluate the performance of the SSM validation approach, since we had previously obtained good results with this method on a different gold standard. The threshold value applied (0.4) was based on [2].

According to the corpora used, run 3 should be used as a baseline for runs 1 and 5, while run 3* should be used as a baseline for runs 2 and 4.

### Semantic similarity using h-index

We used the maximum semantic similarity value of each predicted chemical entity to the other entities identified in the same fragment of text to filter entities incorrectly predicted by the CRF classifiers.

The simUI measure [21] is an edge-based approach to measure the semantic similarity between two classes. Given two classes *c*_1 _and *c*_2_, and the set of their ancestors *asc*(*c*_1_) and *asc*(*c*_2_), this measure is equal to the number of classes in the intersection between *asc*(*c*_1_) and *asc*(*c*_2_) divided by the number of classes in the union of the same two sets:

$$\text{sim UI}\left(c_{1},c_{2}\right)=\frac{\#\left\{t|t\in asc\left(c_{1}\right)\cap asc\left(c_{2}\right)\right\}}{\#\left\{t|t\in asc\left(c_{1}\right)\cup asc\left(c_{2}\right)\right\}}$$

A similar approach for measuring semantic similarity is the simGIC measure [5]. In this case, each ancestor is weighted by its information content (IC), which is a measure of the specificity of a concept. The simGIC is defined as the sum of the IC of the classes in the intersection between *asc*(*c*_1_) and *asc*(*c*_2_) divided by the sum of the IC of the classes in the union of the same two sets:

$$\text{sim}\;\text{GIC}\left(c_{1},c_{2}\right)=\frac{\sum \left\{\text{IC}\left(t\right)|t\in asc\left(c_{1}\right)\cap asc\left(c_{2}\right)\right\}}{\sum \left\{\text{IC}\left(t\right)|t\in asc\left(c_{1}\right)\cup asc\left(c_{2}\right)\right\}}$$

The hierarchical structure of the ontology can be used to quantify the IC of each class. Seco *et al*. [24] proposed an intrinsic IC as a function of the number of subclasses and the maximum number of classes in the ontology:

$$IC\left(c\right)=1-\frac{\text{log}\left(\text{sub-classes}\left(c\right)+1\right)}{\text{log}\left(C\right)}$$

where sub-classes(*c*) is the number of sub-classes of *c *and *C *is the total number of classes in the ontology.

**Figure 7:**
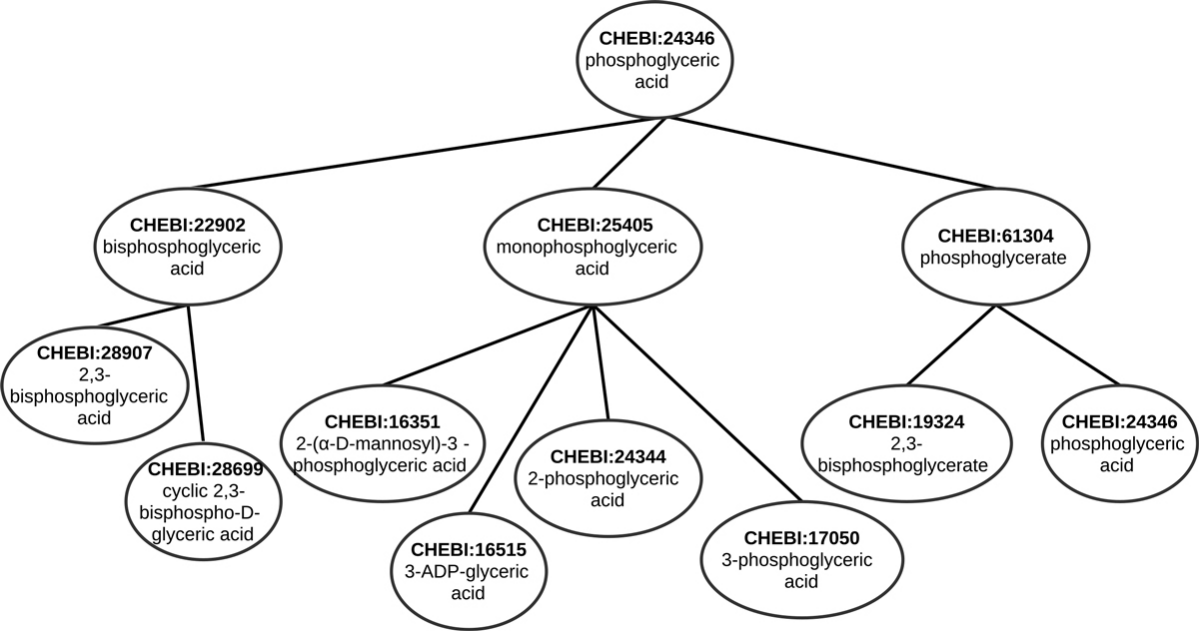
**Section of the ChEBI ontology showing a term (CHEBI:24346) with a h-index of 2, since 2 of its child nodes have at least 2 other child nodes, and the other child node has no more than 2 child nodes**.

Both simUI and simGIC consider every ancestor up to the root. These measures could be improved by selecting only the ancestors that are more relevant in the ontology. We estimated the relevance of a class by adapting the h-index [8] to the ChEBI ontology, defining it as follows: A term has index *h *if *h *of its *N_p _*children have at least *h *children each and the other (*N_p _*- *h*) children have ≤ *h *children each. Figure 7 shows an example of a ChEBI entity (CHEBI:24346) with an h-index of 2. Classes that are leaf nodes or classes that have only leaf nodes as sub-classes have an h-index of 0.

Then, we adapted the simUI and simGIC measures to exclude ancestors with an h-index lower than a certain threshold *α*. Only the ancestors with h-index higher or equal to a are considered for *asc*(*c*_1_) and *asc*(*c*_2_).

$$\text{sim U}{\text{I}}_{h}\left(c_{1},c_{2}\right)=\frac{\#\left\{t|t\in asc\left(c_{1}\right)\cap asc\left(c_{2}\right)\wedge \text{h-index}\left(t\right)\geq \alpha \right\}}{\#\left\{t|t\in asc\left(c_{1}\right)\cup asc\left(c_{2}\right)\wedge \text{h-index}\left(t\right)\geq \alpha \right\}}$$

$$\text{simGI}{\text{C}}_{h}\left(c_{1},c_{2}\right)=\frac{\sum \left\{\text{IC}\left(t\right)|t\in asc\left(c_{1}\right)\cap asc\left(c_{2}\right)\wedge \text{h-index}\left(t\right)\alpha \right\}}{\sum \left\{\text{IC}\left(t\right)|t\in asc\left(c_{1}\right)\cup asc\left(c_{2}\right)\wedge \text{h-index}\left(t\right)\geq \alpha \right\}}$$

Using lower *α *values, fewer ancestors are excluded and consequentially, the similarity values should be closer to the ones obtained with the original measures. As we increase the threshold *α*, only the most relevant classes are considered and the semantic similarity values deviate more from the original.

We performed a similar recognition process to what was used in the competition, but now using the simUI and simGIC similarity measures, and adapted versions which filter ancestors based on their relevance by excluding those with h-index lower than a certain threshold.

Our objective was to improve the overall recall while maintaining high precision values, by better filtering out false positives from the results obtained with our machine learning method. Using our adapted versions of the simUI and simGIC measures, we were able to remove more false positives, for the same number of true positives wrongly removed. In other words, for a fixed recall, we were able to achieve higher precision values.

## Competing interests

The authors declare that they have no competing interests.
